# Self-initiated humour protocol: a pilot study with an AI agent

**DOI:** 10.3389/fdgth.2025.1530131

**Published:** 2025-03-13

**Authors:** Abbas Edalat, Ruoyu Hu, Zeena Patel, Neophytos Polydorou, Frank Ryan, Dasha Nicholls

**Affiliations:** ^1^ Algorithmic Human Development Group, Department of Computing, Imperial College London, London, United Kingdom; ^2^ Faculty of Medicine Centre, Faculty of Medicine, Imperial College London, London, United Kingdom; ^3^ Division of Psychiatry, Faculty of Medicine, Imperial College London, London, United Kingdom

**Keywords:** self-initiated humour, self-attachment, chatbot, virtual reality, wellbeing, self-enhancing humour, self-compassion, emotion regulation

## Abstract

**Introduction:**

Non-hostile humour and laughter have been known for therapeutic benefits in an individual’s mental health and wellbeing. To this end, we evaluated the Self-Initiated Humour Protocol (SIHP), a new type of self-administrable laughter intervention that utilises spontaneous and self-induced laughter. Rooted in the core principles of the Self-Attachment Technique—in which an individual creates an affectional bond with their childhood self as represented by their childhood photo or personalised childhood avatar—SIHP provides an algorithmic framework for individuals to learn to laugh in a non-hostile manner and develop a sense of humour in all possible life contexts. This allows SIHP to be self-administered by interacting with an AI agent.

**Methods:**

An 8-week intervention was conducted with N = 27 adult participants. Exclusion criteria: severe depression or anxiety (PHQ-9 and GAD-7 scores above 15). Participants’ measurements were collected in the areas of wellbeing, use of different humour styles, emotional self-regulation, self-compassion and psychological capital, and analysed to understand any changes over time. Measurements were taken immediately before, after the intervention, and at the 3-month follow-up. Throughout the intervention, participants were required to practise SIHP 20 min a day with the aid of an emotionally intelligent chatbot and their personalised child avatar in virtual reality (VR).

**Results:**

Analysis of results at the 3-month follow-up showed significant improvements in the primary outcome of wellbeing with large effect size (r=0.92), as well as a range of secondary outcomes with large effect sizes, self-compassion (r=0.93), use of self-enhancing humour (d=0.80), and emotion regulation (d=0.87); the results also showed improvement to participant’s psychological capital with moderate effect size (d=0.56).

**Discussion:**

This study shows the potential for the practice of SIHP as supported by an emotionally intelligent chatbot and personalised child avatar to have medium-term positive effects, which should be validated through future randomised trials.

## 1 Introduction

Wellbeing within the general population has been known to influence physical and mental health (1), with low levels of wellbeing correlating with mental health conditions such as anxiety and depression (2). Humour interventions have been studied for their effect on mental health (3, 4). Humour styles are categorized into four: self-enhancing, affiliative, self-deprecating and aggressive, of which the first two styles are non-hostile (5). Non-hostile humour and Duchenne smile and display have been considered the best medicine for physical health and mental health (3, 4, 6). The Duchenne smile and display activate symmetrical, asynchronous, and smooth contractions of two facial muscles at once: the *zygomatic major* muscle in the cheeks that pulls the lip corners upwards and backwards, and the *orbicularis oculi* muscle that surrounds each eye socket and causes wrinkling of the skin at the outer sides of the eyes. While most types of smiles involve contractions of the zygomatic major, only the Duchenne smile, also called genuine smile, involves the involuntary contraction of the orbicularis oculi (4, p. 179).

In addition, a leading review article on mental illness argues that anhedonia or the inability to experience joy and interest in activity is the common denominator of a host of mental conditions including depression, anxiety and post-traumatic distress disorder, and advocates a paradigm shift from focusing on the alleviation of negative symptoms to targetting enhancement of positive affect (7). Humour interventions, we may infer, can be beneficial both in non-clinical and clinical populations.

Existing laughter interventions can be broadly summarised into two paradigms (8): (a) Spontaneous or genuine, humour-based laughter, usually requiring a constant flow of jokes, and (b) Self-simulated, intentional or feigned Yoga laughter, which has its roots in Buddhism.

A recent systematic review of laughter interventions has investigated the impact of laughter on recovery from psychological stress using the Connectedness, Hope, Identity, Meaning and Purpose and Empowerment (CHIME) framework (9). It examines a host of comedy interventions of type (a) above, and concludes:

“Overall, the studies provided limited detail about the content of the comedy interventions or exactly how participants engaged with the multi-faceted dimensions of comedy in the interventions. Instead, the studies focused on how results post-intervention compared to measures identified pre-intervention, generally omitting details of what exactly happened in between. As a result, the comedy interventions remain largely a ‘black box.”’

We note that the review included five studies that evaluated the “7 Humour Habits” programme (10), which teaches humour skills based on the seven steps: (1) Surround yourself with humour. (2) Cultivate a playful attitude (view life through a lighter lens). (3) Learn to laugh at yourself. (4) Practice telling jokes, (5) Find humour in adversity, (6) Encourage humour in others (foster a humorous environment). (7) Create humour.

The Self-Initiated Humour Protocol (SIHP) (11) is a new self-administered, algorithmic framework for learning to laugh in a non-hostile manner in all circumstances one might find in life to enhance wellbeing. SIHP synthesises the spontaneous/humorous paradigm with the intentional/self-induced paradigm of laughter. In comparison with the “7 Humour Habits,” SIHP formulates specific rules to create non-hostile laughter and humour.

SIHP provides a set of rules for seeing the funny side of life in all contexts by using the main theories of humour, in particular the Superiority theory, the Incongruity theory and the Play or Evolutionary theory of laughter (11, 12). Other theories of humour can be considered as a particular instance of these theories (13). In SIHP, the Superiority theory is employed in a self-reflexive way; for example, the individual is encouraged to laugh as a sign of victory after completing any mundane daily task, such as washing up and cleaning. This provides the self with a sense of superiority compared to their previous self, which did not express any joy in doing these tasks.

In addition, SIHP incorporates, what has been called, the *Perspective* theory of humour based on two well-known quotes by Charlie Chaplin, a universal icon of modern comedy, namely: “Life is a tragedy when seen in close-up, but a comedy in long-shot” and his proposed mechanism for turning tragedy to comedy, “To truly laugh, you must be able to take your pain, and play with it.”

Rather than relying on comedy and jokes or just intentional laughter, as in most existing laughter interventions, SIHP employs the individual’s mindset and their interpretations of the world, including their inner world, to generate humour using its set of rules based on these theories of laughter, which give the rationale for the underlying humour in these rules. The aim is to help the individual to become a humorist. This implies that SIHP can be self-administered, and, crucially, its practice does not require participation in a group as is usually the case with existing humour interventions. Indeed, while most laughter takes place with one or more other individuals—which is consistent with the Evolutionary theory of laughter as a play signal in games of higher primates—it is known that some laughter does take place by human individuals on their own (5, 14).

SIHP challenges the deep-seated beliefs, usually entrenched in childhood development (4), that humour and laughter are inconsistent with encountering problems, setbacks, misfortunes and tragedies. It argues that we can be playful in almost all contexts, including after absorbing the shock of a misfortune or tragedy. The laughter protocol adopts a developmental approach and is embedded within the Self-Attachment Technique (SAT) (15–17). SAT is a self-administered psychological intervention, rooted in attachment theory (18–21), which aims to re-raise our childhood self into social and emotional maturity. SIHP expands upon the basic set of laughter exercises from SAT and frames its exercises with the core principles of SAT, i.e., as a two-actor role-playing scenario consisting of an individual simultaneously taking the roles of a “good enough” care-giving parent and a care-seeking child. The self-administered nature of SIHP makes it suitable for its delivery to be supported through digital technologies, such as VR (22, 23) and emotionally intelligent chatbots (24, 25).

Conversational agents, commonly referred to as chatbots, present an accessible, scalable and personalisable platform (26, 27) for digital healthcare (28), in particular, for the deployment of psychotherapeutic interventions (29, 30) through conversing with the user to provide protocol-relevant support and recommendations. Previous works in chatbot-based SAT (24, 25) produced web-based chatbots that utilised rule-based conversation flows, using a combination of open-text and fixed-text inputs to provide exercise recommendations and guidance to the user. Based on such an approach, the outcomes of our experiments indicated that the use of chatbots was beneficial to user engagement with the protocol (24). The addition of humour carries the potential to bring further improvements to the SAT-based SIHP intervention. Recent works have explored the benefits of adding humour into chatbots in non-healthcare settings such as in teaching (31), customer service (32) and personal assistants (33). Although the use of humour in chatbots for mental healthcare has not yet been thoroughly explored, recent works have nonetheless investigated the integration of humour into chatbots for a variety of interventions (34), including Behaviour Change Technique (35, 36) and Motivational Interviewing (37, 38), all reporting similar user preferences towards more humorous conversational agents, and the resulting improvement in user engagement.

Based on the findings of the aforementioned experiments, we conducted an 8-week human trial in the adult population for the SIHP protocol. We hypothesised that practising SIHP, aided by digital technologies such as an emotionally intelligent chatbot and VR, can induce improvement in an individual in the primary area of wellbeing and secondary areas of self-compassion, use of self-enhancing humour and psychological capital. We have also investigated user perception of the chatbot in guiding users in the practice of SIHP similar to previous works (30). Our study was granted ethical approval by the Imperial College Research Ethics Committee (ICREC/SETREC reference 22IC7536). All experiments were performed in accordance with relevant guidelines and regulations, and informed consent was obtained from all participants prior to the start of the study.

## 2 Materials and methods

### 2.1 Study design

We designed and conducted an 8-week pilot study to ascertain the feasibility of the protocol, along with its effects on a multitude of factors. Our study hypothesis was that the practice of the protocol through an 8-week intervention can enhance the user’s wellbeing (primary outcome), as measured using the PERMA-profiler (39) through measured dimensions of Positive emotions, Engagement, Relationships, Meaning and Accomplishment. We include within our hypothesis the enhancement of the following secondary outcomes: humour styles as measured by the Humour Styles Questionnaire (HSQ) (5), emotional self-regulation as measured by the Emotion Regulation Questionnaire (ERQ) (40), self-compassion as measured by the Sussex-Oxford Compassion for the Self Scale (SOCS-S) (41), and psychological capital (problem-solving) as measured by the revised Compound Psychological Capital Scale (CPC-12R) (42).

Adult participants were recruited by advertising through in-person channels such as word-of-mouth and posters, and online via adverts posted to the social media platform Facebook. Screening was conducted using the GAD-7 (43) and PHQ-9 (44) questionnaires to ensure participants met our inclusion criteria below “moderately severe” depression and anxiety measures (below 15 PHQ-9 and GAD-7 scores) in order to participate in the study.

**Figure 1:**
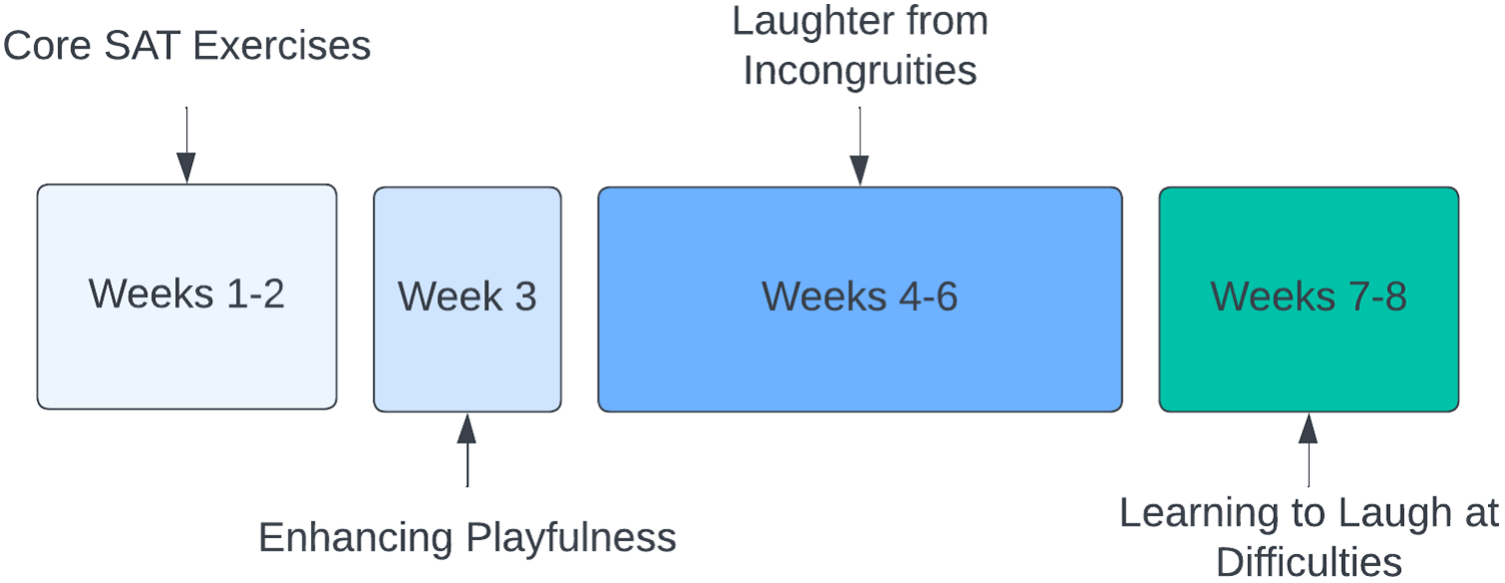
Schedule of exercise types by week.

A subset of the SIHP exercises was released to the participants each week through online sessions held remotely via Zoom, during which participants were given the opportunity to comment on and ask questions about the exercises from the past week before being introduced to the exercises for the coming week. Presentations on the exercises were delivered by AE, who originally pioneered both SAT and SIHP. Recordings of the online sessions and exercise materials were uploaded immediately afterwards. Topics covered in each week of the 8-week intervention are shown in Figure 1; more details on the exercises can be found in Section 2.2. Participants were expected to independently practise the exercises for at least 20 min daily, noting their daily progress in a diary. In the event that participants were unable to attend the meeting at the scheduled time, recordings of the meetings were made available to watch in their own time. All study materials throughout the intervention were made available digitally via a study information webpage, accessible by participants using their unique ID. All participants were given the same protocol documents, which remained unchanged for the duration of the intervention.

### 2.2 Self-initiated humour protocol

We now detail the principles of the self-initiated humour protocol. SIHP exercises are framed based on the principles of SAT, with core SAT exercises being practised before SIHP exercises, reflecting the protocol’s developmental approach to learning as humorous. At the outset, participants are asked to choose a non-materialistic, socially useful, noble life goal. The 8-week protocol is seen as empowering them with the means to attain their goal and overcome all obstacles and problems on the way.

The core SAT exercises seek to establish a compassionate and affectional bond between the adult self and the child self, including visualising the child self using childhood photographs or VR child avatar, imaginatively interacting with the child self through singing, dancing and verbal reassuring, culminating in the adult self vowing to look after the child. This allows the individual to externalise their negative emotions onto the child self in times of distress and to offer comfort to the distressed child, which means to take care of themselves.

SIHP exercises, beyond core SAT, can be broadly partitioned into two groups; the first group of initial exercises aims to promote a playful mode within an individual to prepare them for laughter, and the second group of laughter exercises provide context and a trigger for non-hostile Duchenne laughter. Each SIHP laughter exercise is grounded on at least one of the four main theories of humour discussed previously, which are abbreviated as: *Superiority*, *Incongruity*, *Play* and *Perspective*. Each exercise is also carried out in the context of an interaction between the adult and child, as the adult self invites the child to undertake the exercise, an invitation which plays a key role when trying to laugh off difficulties, upsets and misfortunes. As explained in (11), this can be compared to Freud’s attempt to explain the mindset of the humorist in expressing dark humour as a suggestion made by the superego to the ego (45), which we will critically discuss in Section 4.

A detailed description of SIHP exercises grouped by theme is as follows. In each case, we state at the end which theory or theories the exercise is based on.

1. **Playful mind/face.** We engage our body and mind in a playful mode in preparation for laughter. We learn to be playful in mind by imaginatively exaggerating or reversing our established beliefs, without necessarily abandoning them. Likewise, we can prepare our facial muscles for laughter by purposefully moving and relaxing the muscles around the mouth and eyes. (*Play*)
2. **Contrasting views.** With the help of Gestalt images (i.e., a vase), we learn to be cognizant of changes in our perception and frame this in a humorous manner as a trigger for Duchenne laughter. (*Incongruity*, *Play*)
3. **Self-glory.** We learn to recognise and laugh at simple, daily accomplishments such as completing household chores. (*Superiority*, *Play*)
4. **Feigning laughter.** We intentionally simulate Duchenne laughter (yoga laughter), without any underlying humorous context. (*Superiority*, *Play*)
5. **Self-laughter.** We learn to laugh in a non-hostile manner at our errors, blunders, mistakes and faults. (*Superiority*, *Incongruity*, *Play*)
6. **Personal laughter brand.** We can further expand our capacity for laughter by creating our own brand of laughter through varying combinations of vowel sounds and rhythms. This also provides us with a gentle form of laughter which consumes a minimal amount of energy and can thus be sustained over long periods of time. (Superiority, *Play*)
7. **Incongruous world**. We learn to laugh without hostility at the inconsistencies, contradictions, contrasts and dissonance we perceive in the world, always laughing at situations, systems, circumstances and contexts rather than aggressively at people. (*Superiority*, *Incongruity*, *Play*)
8. **Incongruity between our expectation and reality.** While it is natural to experience negative emotions as an immediate reaction to a setback, we learn to try to make a shift as soon as possible after assimilating the shock and laugh at the incongruity that our expectations are violated, thereby enhancing our resilience. (*Superiority*, *Incongruity*, *Play*)
9. **Incongruity within.** We look out to recognise any incongruity, inconsistency and contradiction in our behaviour and thoughts, which we can laugh at in a non-hostile way, an exercise which can also make us wiser. (*Superiority*, *Incongruity*, *Play*)
10. **Laughing at short-term or long-term difficulties.** We bear in mind that encountering and overcoming problems and suffering can make us stronger, as exemplified in the quote: *What does not kill me makes me stronger*. We therefore learn to laugh, in due course, at current misfortunes/circumstances or past/long-term difficulties, knowing that they can make us stronger. (Perspective, *Superiority*, *Incongruity*, *Play*)

### 2.3 Participants

**Figure 2:**
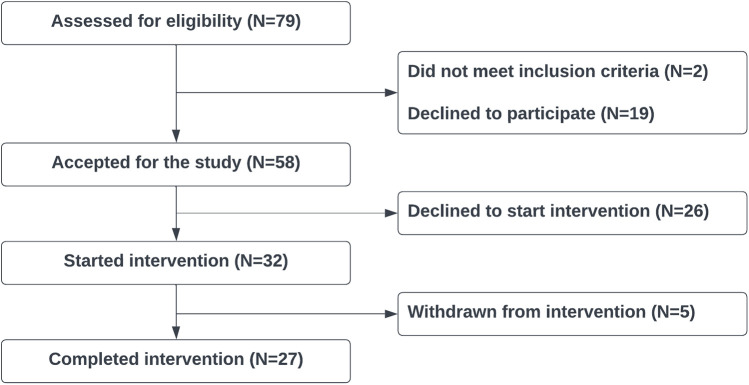
Consort diagram.

We conducted recruitment and carried out the study in accordance with the methodology outlined in Section 2.1. We began the intervention with 32 participants, of which 5 withdrew from the intervention between weeks 3 and 6 due to changing personal circumstances (15.6%), as a result, 27 participants (14 females, 13 males) completed the 8-week intervention. The consort diagram shown in Figure 2 illustrates the cohort size in more detail. Our cohort size was informed by a power analysis (46, 47) conducted prior to the start of the study with (i) an expected moderate (medium) effect size of 0.65 [informed by the results of the VR-based SAT study (23)], (ii) a statistical power of 0.8 (β=0.2), and (iii) an accepted significance of p<0.05. Combined with an expected dropout rate of up to 20%, we yield the minimum accepted sample size of *N* ≥ 25.7, below our cohort size of *N* = 27.

Participants were required to complete up to 11 questionnaires prior to the start of the intervention and complete a daily diary summarising their experiences with their 20 min daily exercises. Some individuals, who showed an initial interest in the advert for the study in the social media platform Facebook, declined to take part subsequently, citing the high commitment of time and effort as the reason.

We report only on the data collected from the 27 participants who completed the intervention. Of the 27 participants, 2 declined to provide feedback for the chatbot, citing insufficient use across the intervention. We recorded weekly session attendance and daily diary completion throughout the 8-week intervention as an indication of participant compliance. On average, each participant attended 5.81 sessions (SD = 2.39) out of nine (8 exercise sessions and a concluding session) and completed 40.19 diary entries (SD = 16.64) out of 56 days of the intervention. We ensured participants’ overall compliance by observing the diaries.

### 2.4 Childhood avatar and VR app

After being selected for the study, each participant provided their favourite childhood photo which was used to create their childhood avatar in a similar way to the previous VR-based SAT study (23). Participants were provided with a personalised mobile VR app to interact with their childhood avatar that can be animated in seven basic emotional states and dance with their favourite love song. This VR platform (23) allowed participants to conduct the core SAT exercises, namely to create a compassionate connection and an affectional bond with their childhood self, and learn to enhance positive and reduce negative affects by projecting their emotions to the avatar. The use of childhood avatars—instead of childhood photos as in the first clinical SAT study (48)—had shown some good potential in three short user evaluations (22, 49, 50) and subsequently demonstrated promising results for enhancing wellbeing and self-compassion in a proper 8-week human trial (23).

### 2.5 Emotionally intelligent AI agent

We aimed to develop an emotionally intelligent chatbot to guide the user in practising SIHP. Given the problem of possible toxicity and hallucination in large language models (LLMs), we decided to employ a completely safe rule-based chatbot that is designed to be empathetic, non-repetitive, engaging and humorous in its responses, while being able to detect the emotion of the user. To this effect, we curated a dataset of the rule-based chatbot responses and an AI platform based on the dataset, as we will explain in the next two subsections.

#### 2.5.1 Chatbot response corpus

We collected and curated a dialogue corpus consisting of 122 base responses corresponding to each conversation state of the chatbot. For the purposes of enabling variable conversation whilst maintaining safety and predictability, we augmented each of the 122 base responses corresponding to a conversation state with responses rewritten by participants recruited from the crowdsourcing platform Prolific^1^ and manually inspected each response to filter out inappropriate responses (i.e., offensive, unsafe). In line with our aim to make conversations with the user engaging, empathetic and humorous, we requested rewritings from all surveys to be polite, empathetic and fluent with the optional inclusion of light-hearted humour if deemed appropriate by the participants. Examples were provided to guide the participants as to the appropriate styles of responses. Participants were free to be imaginative, provided that the rewriting remained semantically consistent with the original response, or that their response otherwise corresponded to the user’s utterance.

In order to improve the purposes of consistency across participants’ responses, we grouped base responses by theme (humorous, general, scenario-based) such that each participant focuses on a single thematic task. Our decision to split humorous and non-humorous responses in rewritings between participants was primarily to avoid the induction of humour in inappropriate manners that may cause distress to the user, i.e., the base response “Has there been any long-term difficulty in your life that may be causing this negative feeling?” was deemed inappropriate for a humorous rewriting. Assigning a subset of responses to each participant also serves as a quality control measure to limit the impact of any potential bias on the whole corpus. In addition to verifying the thematic and tonal criteria when manually inspecting each response, we made small adjustments to correct grammar, punctuation and fluency where necessary.

We further augmented the responses provided by 54 crowdsourced workers with responses from 67 volunteers to improve the balance in the number of re-writings per response and supplement any discarded responses. In total, we collected 2,507 total responses, with at least 12 candidate responses for each turn of conversation. Our volunteers consisted of 69 females and 52 males predominantly aged between 18 and 24.

#### 2.5.2 Online chatbot platform

A web-based chatbot platform was provided to the user, which offered exercise recommendations and guidance based on the user’s emotional state and past exercise completion. The platform was designed to allow for easy access to help in practising each exercise. Separate conversation flows were devised based on the user’s detected emotion; the conversation flow in the case of a positive emotional state aims to further enhance the user’s positive emotions; conversely, the chatbot can guide the user in exploring potential causes of their negative emotions and suggesting appropriate SIHP exercises to help them overcome their negative emotions. Following examples from previous works of a similar nature (24, 25, 37) we implemented a predefined series of conversation states, with variation in chatbot responses available for a given conversation state so as to prevent the conversation becoming repetitive across multiple impressions. The basic conversation flow can be described as follows:

• The conversation begins with the chatbot greeting the user and asking how they are doing. The user is able to provide open-text responses to describe recent events and their effects on their mood.
• If the user is detected to be in a positive emotional state, the chatbot guides the user towards either experiencing a new SIHP exercise or practising an existing exercise.
• If the user is detected to be in a negative emotional state, the chatbot supports the user by providing suggestions for how to tackle their negative emotions, beginning with identifying the cause, and then recommending appropriate corresponding SIHP exercises, which can also be a core SAT exercise.
• At any given point the user has the option to select a SIHP exercise directly.
• Once selected, the chatbot will guide the user through practising an exercise.
• At the end of each session, the chatbot will again check the user’s emotional state, providing appropriate responses before ending the session.

Humorous discourse is provided at fixed points in the conversation (35), with user response employed to tune the frequency at which a humorous utterance is presented in order to allow user preference to mitigate the issue with subjectivity in humour (34). More details on the chatbot design can be found in the conversation flow diagrams and example conversations included in the Supplementary Material. Due to data protection and user privacy reasons, we did not collect dialogue transcripts.

To allow for variety in conversation over repeated interactions with the chatbot, we followed previous work (24) and collected multiple human-written responses for each turn of conversation (Section 2.5.1). During a conversation, the chatbot selects an appropriate response based on a weighted score measuring: empathy exhibited $\tilde{E}$, humorous tone $\tilde{H}$, sentence fluency $\tilde{F}$ and novelty to conversation $\tilde{N}$. Dedicated retrieval functions $O_{\mathrm{pos}}$ and $O_{\mathrm{neg}}$ prioritise different attributes depending on whether the user is in a positive or negative emotional state, i.e., should the user be detected to be in a negative emotional state, the chatbot would prioritise retrieving empathetic responses over humorous ones:

(1) $$O_{\mathrm{pos}}=0.1\cdot \tilde{E}(s)+0.4\cdot \tilde{H}(s)+\tilde{F}(s)+6\cdot \tilde{N}(s)$$

(2) $$O_{\mathrm{neg}}=0.4\cdot \tilde{E}(s)+0.1\cdot \tilde{H}(s)+\tilde{F}(s)+4\cdot \tilde{N}(s)$$

The weighting of each component, in the Expressions 1, 2, was deduced experimentally to optimise the quality of retrieved utterances.

In order to compute the humour score used in response retrieval, we tasked three human annotators with labelling a subset of the response corpus consisting of 1,109 examples for the presence of humour, with the majority label being used (Krippendorff’s α = 0.57). We formulated the humour detection task as a binary classification task and fine-tuned a RoBERTa-base (51) model on an 80-5-15 train-val-test split of the labelled dataset. Our approach achieved an Accuracy of 97% and a Macro-F1 score of 97%, significantly improving upon the logistic regression (+18%) and naive Bayes (+21%) approaches proposed by previous work (24). Additionally, we experimented with fine-tuning a BERT-base (52) but found it to be slightly outperformed by the RoBERTa model (+2%), we therefore chose to use RoBERTa in computing $\tilde{H}(s)$ for each sentence s within our response corpus. For empathy $\tilde{E}(s)$, fluency $\tilde{F}(s)$ and novelty $\tilde{N}$ components, we leveraged the models used in the previous work (24).

At the end of the 8-week intervention, we asked participants to rate on a 1-5 Likert scale the tone (humour, empathy, politeness) of the chatbot and the quality of conversation (engagement, flow, accessibility), as well as provide qualitative comments (Section 3.3).

### 2.6 Statistical analysis

For each of the 5 reported outcomes (1 primary, 4 secondary), we analysed the participant responses collected at the end of the 8-week intervention (Post) and at the 3-month follow-up (Follow-up), against the responses collected immediately prior to the start of the intervention (Pre). For each outcome, we first tested the difference between pre-intervention and post-intervention results for normality (Shapiro-Wilk).

In the event that the normality condition was satisfied, we calculated Cohen’s d multiplied by Hedge’s correction factor (53) (Equation 3), using the t-test for statistical significance:

(3) $$\text{Hedge's correction factor}=1-\frac{3}{4(n_{1}+n_{2})-9}$$

where $n_{1}=n_{2}=27$ are respectively the sample sizes for the measured cohort at the pre-intervention stage, and the post-intervention or 3-month follow-up stages. The effect size for the updated value of d is considered small for 0.2≤d, medium for 0.5≤d and large for 0.8≤d.

If, however, normality was not satisfied, we conducted the Wilcoxon signed-rank test for statistical significance and calculated the Matched-pairs rank-biserial correlation (r) (54) as a measure of effect size. The effect size, in this case, is considered small for 0.1≤r, medium for 0.3≤r and large for 0.5≤r. In both cases we apply Bonferroni correction (55) to the p-value with $N_{\mathrm{tests}}=18$ to use p<0.0028 for our tests of significance.

## 3 Results

### 3.1 Main analysis

The statistical analysis of the results (*N* = 27) for the primary outcome and 4 secondary outcomes are shown in Table 1.

**Table 1:** Statistical analysis for primary and secondary outcomes for (*N* = 27) subjects.

Outcome	Comparison	Measure	*t*-value	ES(d)	*Z*-value	ES(r)	*p*-value
PERMA	Pre/post				4.41	0.97	<0.001
	Pre/follow-up				4.19	0.92	<0.001
HSQ	Pre/post	Affiliative	0.20	0.05			0.815
		Self-enhancing	3.52	0.94			<0.001
		Aggressive	0.32	0.09			0.728
		Self-defeating	0.82	0.22			0.100
	Pre/follow-up	Affiliative	0.20	0.05			0.822
		Self-enhancing	3.00	0.80			<0.001
		Aggressive	−0.18	−0.05			0.822
		Self-defeating	1.42	0.38			0.006
ERQ	Pre/post	Reappraisal	3.62	0.97			<0.001
		Suppression	−0.81	−0.22			0.092
	Pre/follow-up	Reappraisal	3.24	0.87			<0.001
		Suppression	0.09	0.02			0.842
SOCS-S	Pre/post		4.10	1.10			<0.001
	Pre/follow-up		3.46	0.93			<0.001
CPC-12R	Pre/post		2.73	0.73			<0.001
	Pre/follow-up		2.10	0.56			<0.001

*t*-value, *t*-test statistic; ES(*d*), effect size (unbiased Cohen’s d average); *Z*-value, *Z*-statistic of the Wilcoxon signed-rank test; ES(*r*), effect size (matched-pairs rank-biserial correlation); *p*-value, significance value.

The analysis showed significant improvement in PERMA (39) scores, measuring participant wellbeing, between pre-test (median = 6.69) and post-test (median = 8.38) with large effect size Z=4.41, p<0.001, r=0.97. This improvement was maintained at the 3-month follow-up (median = 7.94), with a large effect size at Z=4.19, p<0.001, r=0.92.

The results for the HSQ (5) showed significant improvement in the self-enhancing humour style between pre-test (median = 35.0) and post-test (median = 43.0) with large effect size t(26)=3.52, p<0.001, d=0.94. This significant increase in self-enhancing humour style is maintained at the 3-month follow-up (median = 42.0) with large effect size t(26)=3.00, p<0.001, d=0.80.

Significant results were observed for the reappraisal dimension of the emotion regulation process in the ERQ (40) measurement between pre-test (median = 4.50) and post-test (median = 5.67) with large effect size t(26)=3.62, p<0.001, d=0.97. This increase is maintained at the 3-month follow-up (median = 5.83) with large effect size t(26)=3.24, p<0.001, d=0.87.

A significant increase in self-compassion was observed in the results for the SOCS-S (41) measurement, between the pre-test (median = 71.0) and post-test (median = 85.0) with large effect size t(26)=4.10, p<0.001, d=1.10. This significant increase is maintained at the 3-month follow-up (median = 84.0) with large effect size t(26)=3.46, p<0.001, d=0.93.

Lastly, we observe a significant increase in participants’ problem-solving capabilities from the results of the CPC-12R (42) measurement between pre-test (median = 55.0) and post-test (median = 61.0) with medium effect size t(26)=2.73, p<0.001, d=0.73. This increase is maintained at the 3-month follow-up (median = 60.0) with medium effect size t(26)=2.10, p<0.001, d=0.56.

### 3.2 Exploratory subgroup analysis

Our sample population included a mixture of participants from the non-clinical (with GAD-7 and PHQ-9 scores below 5) and subclinical (with GAD-7 and PHQ-9 scores at or above 5 and below 15) populations. Based on our inclusion criteria and the thresholds defined by our screening questionnaires (43, 44), we identified participants with either PHQ-9 or GAD-7 scores above “minimal” (at or above 5) to obtain a subclinical subgroup of (*N*  = 17) participants. Although we did not recruit explicitly with the intent to evaluate the subclinical population, we nonetheless conducted exploratory analysis into the results of this subgroup. The full results are shown in Table 2. The results of the subclinical subgroup showed significant improvements in our primary outcome of participant wellbeing (PERMA) between the pre-test (median = 5.81, std = 1.73) and post-test (median = 8.06, std = 1.28) with large effect size (t(16)=4.10, p<0.001, d=1.37). Similarly, a significant increase was observed between the pre-test and follow-up (median = 7.81, std = 1.77) with large effect size (Z=3.62, p<0.001, r=1.0). The range of secondary outcomes also showed similar trends to the main cohort, with all significant changes in the main cohort also being observed within this subgroup. Due to the overlapping samples between the subgroup and the full cohort, we do not make any direct comparison of the effect sizes, though we note the results from the subclinical subgroup as quite promising.

**Table 2:** Statistical analysis for primary and secondary outcomes for (*N* = 17) subjects from the subclinical depression subgroup.

Outcome	Comparison	Measure	*t*-value	ES(d)	*Z*-value	ES(r)	*p*-value
PERMA	Pre/post				4.10	1.37	<0.001
	Pre/follow-up		3.62	1.0			<0.001
HSQ	Pre/post	Affiliative	−0.19	−0.06			0.853
		Self-enhancing	3.19	1.07			<0.001
		Aggressive	0.15	0.05			0.864
		Self-defeating	0.33	0.11			0.528
	Pre/follow-up	Affiliative	0.22	0.07			0.822
		Self-enhancing	3.15	1.06			<0.001
		Aggressive	−0.21	−0.07			0.818
		Self-defeating	1.21	0.41			0.067
ERQ	Pre/post	Reappraisal	3.93	1.32			<0.001
		Suppression	−0.52	−0.18			0.332
	Pre/follow-up	Reappraisal	3.81	1.30			<0.001
		Suppression	0.11	0.04			0.813
SOCS-S	Pre/post				3.62	1.0	<0.001
	Pre/follow-up		4.11	1.38			<0.001
CPC-12R	Pre/post				3.62	1.0	<0.001
	Pre/follow-up		2.34	0.81			<0.001

*t*-value, *t*-test statistic; ES(*d*), effect size (unbiased Cohen’s d average); *Z*-value, *Z*-statistic of the Wilcoxon signed-rank test; ES(*r*), effect size (matched-pairs rank-biserial correlation); *p*-value, significance value.

### 3.3 Chatbot evaluation

In addition to the results of the inventories shown in Section 3.1, we collected quantitative results of participants experience with the rule-based chatbot outlined in Section 2.5.2, as well as qualitative feedback identifying areas of improvement.

**Figure 3:**
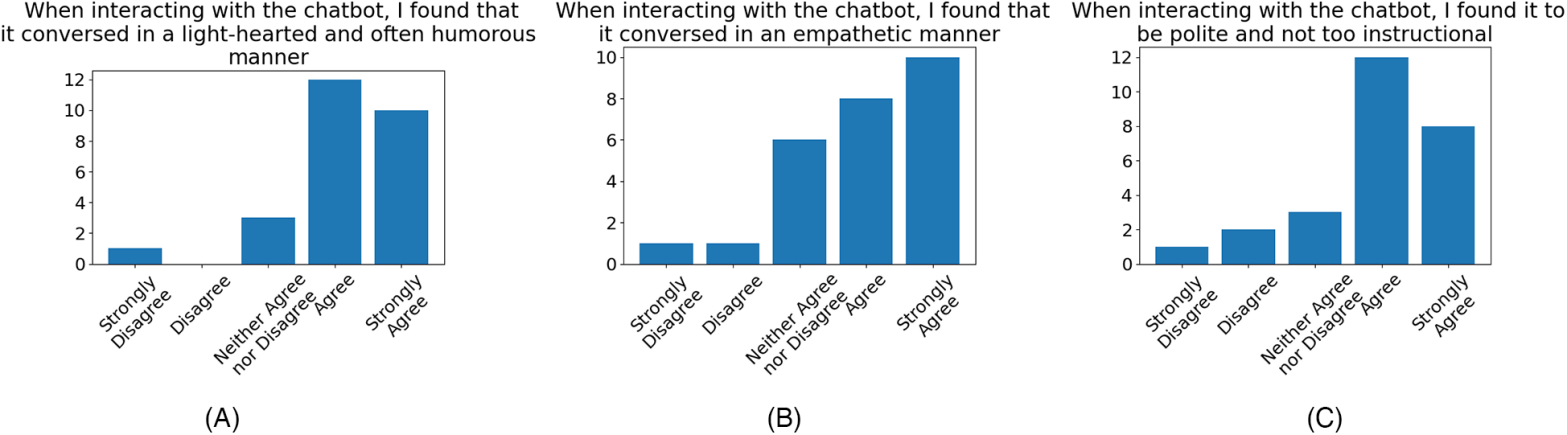
Participant levels of agreement evaluating the tone and style of chatbot utterances, that chatbot generally conversed in a: **(A)** light-hearted and humorous manner (84.62% agreement, 3.85% disagreement), **(B)** empathetic manner (69.23% agreement, 7.69% disagreement), **(C)** polite and not-too-instructional (76.92% agreement, 11.54% disagreement).

Participant responses regarding the tone and style of the chatbot utterances (Figure 3) were generally positive. A few participants expressed disagreement over the tone of the utterances, with one participant expressing “Strong Disagreement” over all given evaluation dimensions. We note from qualitative feedback provided that this sentiment is mainly due to a combination of the subjective nature of certain humorous phrases, which may be misinterpreted under different contexts, and the usage requirement of the chatbot over a long period of time causing some phrases to seem repetitive. This is a known limitation of retrieval-based chatbots. Whilst the predictability of the chatbot provides strong safety guarantees against any toxic or harmful speech, future work should nonetheless address the issues arising from repeated phrases. The issue surrounding the subjectivity of certain humorous responses highlights a requirement for the personalisation of chatbot tone, such as allowing the user to adjust the frequency of quips.

**Figure 4:**
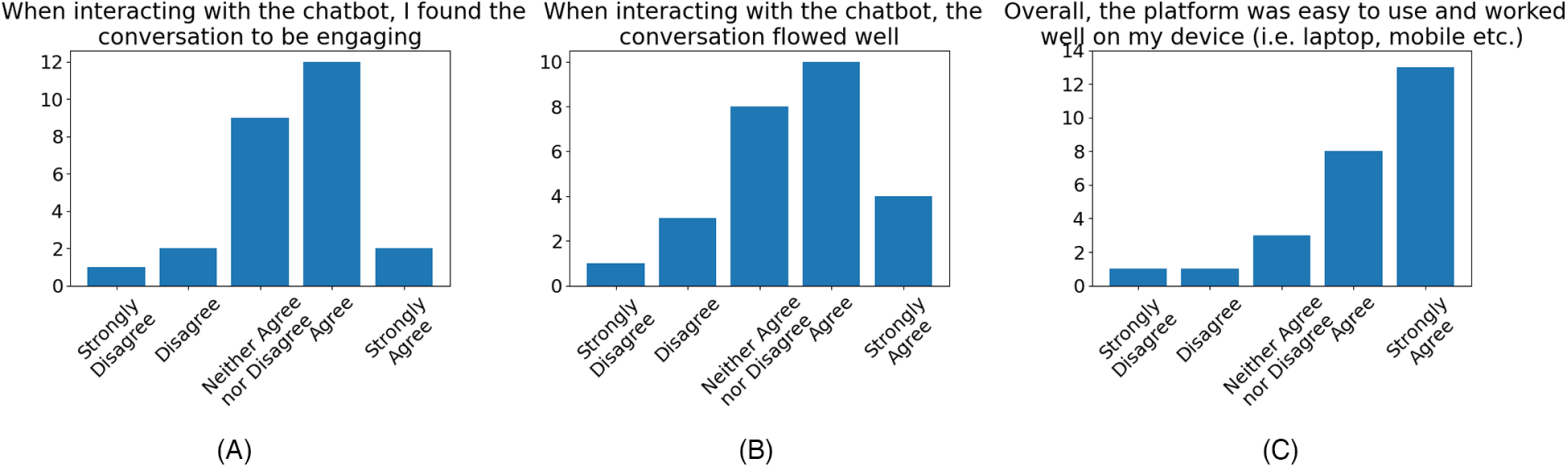
Participant agreement evaluating the quality of conversation with the chatbot, that the: **(A)** conversation was engaging (53.85% agreement, 11.54% disagreement), **(B)** conversation flowed well (53.85% agreement, 15.38 disagreement), **(C)** chatbot was easy to use (80.77% agreement, 7.69% disagreement).

Participant levels of agreement over the quality of the conversation (Figure 4), whilst showing general agreement, is much less positive than the aforementioned reception to chatbot tone. Feedback provided often cited the rigid conversation flow and lack of utterance diversity as a source of consternation. However, participants generally responded positively to the platform’s ease of use.

**Figure 5:**
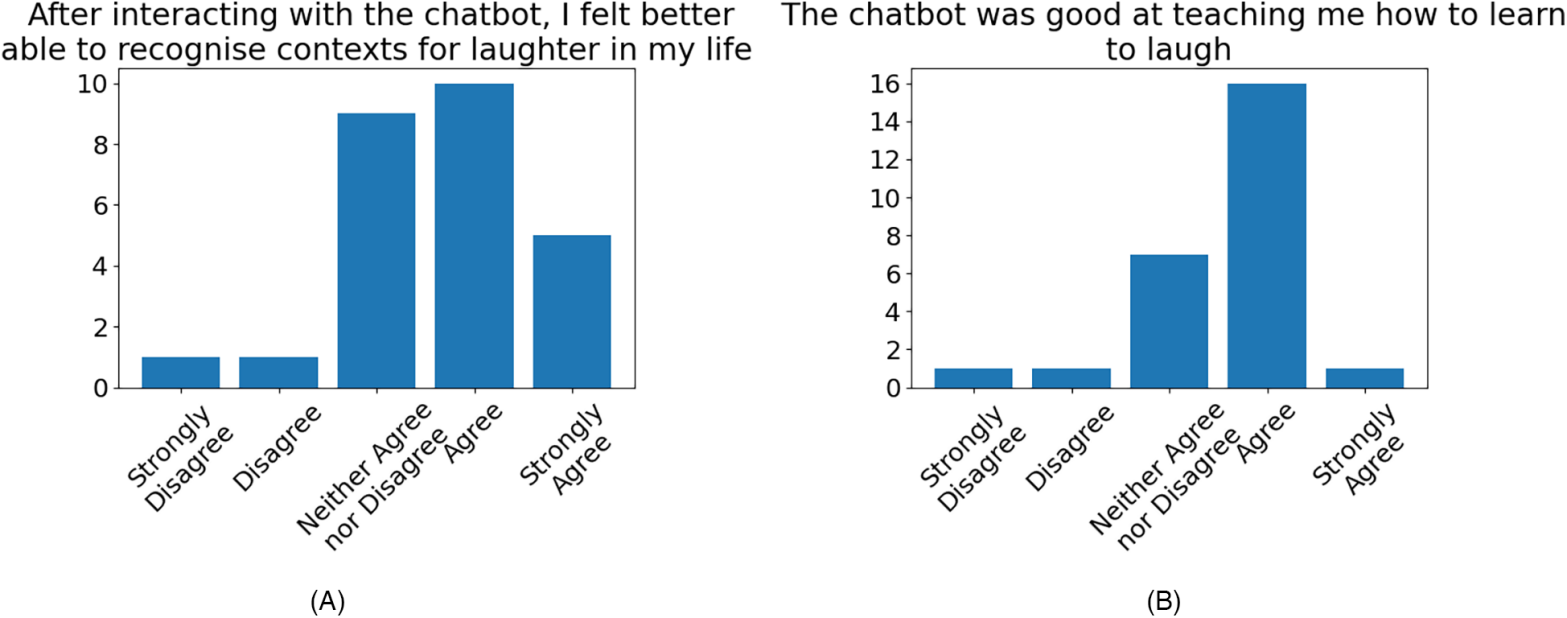
Participant agreement over the efficacy of the chatbot in delivering the protocol, for: **(A)** recognising contexts for laughter (57.69% agreement, 7.69% disagreement), **(B)** teaching the user to learn to laugh (65.38% agreement, 7.69% disagreement).

Participants’ sentiments on the efficacy of the chatbot as a platform for delivering the protocol were generally positive (Figure 5).

### 3.4 Participants’ feedback

We present here some of the feedback from the participants at the end of the course, which we will use to discuss the results of the study in Section 4. Minor edits were made from the transcript of the original spoken quote to aid readability and remove disfluencies, but care was taken to preserve as much of the original speaker’s tone as possible. The complete list of quotes is provided in the Supplementary Material.

1. “I [am] particularly connected with my avatar which I continue using; and sometimes I [cope] in situations, where I can’t necessarily deal with my adult self, by reaching out for the image of the avatar.”
2. “…The bot has been very fun to use and everything else that has been the last eight weeks, an incongruous journey. I learned to laugh at the incongruous in life… It has been a really good experience.”
3. “It really [was] a transformative experience. It changed my view of my childhood experiences and created a lot of self-compassion, and doing the laughter exercises [was] really fun, particularly starting with creating laughter with no reason whatsoever and I found it quite contagious…..”
4. “I find it particularly helpful in looking at my errors of judgement, misperceptions, incongruities and so on… While previously I would try and argue with myself or with the others to try and find an acceptable position, I now discovered that in fact I could completely do none of that and just laugh at it.”
5. “…Going through the exercises seemed very interesting and I really wanted to try it out. And I’m actually very glad that I did. I think it helped me reconnect to my childhood self to bring back a bunch of memories, whether it be good or bad, and just be able to empathise with myself a little bit more and kind of see myself for who I am and know that these are the experiences that shaped me to who I am now. And I think the aspect of humour and laughing […] is a skill that I’m slowly learning to develop because of this. But I definitely hope to take this forward in many future instances in my life, both personal, work and otherwise.”
6. “Being able to reconnect with my younger self, was really helpful. Surprisingly, [in] the last two weeks, I got to relive a past trauma exactly as before, and I was impressed that I took it so lightly this time. And… I was really being humorous as much as I can and sarcastic, but not in a bad way. And I was accepting the outcomes as is. [Previously] it was really a very dramatic experience in my family’s life. So I feel it’s effective, it’s working. I know I’m not yet humorous, but at least I have the tools and I just need to remind myself and keep on repeating it.”
7. “…My young avatar was a bit of a revelation and though there is still work to be done there, it was inspirational and it helps me take a different perspective on my earlier life and somehow reconcile myself to whatever went on. But it is also casting it really in a different light… It is good to know that [even though] there are traumas there… [one can] trigger a laughter, just by choosing the right vowel, and it is a light-hearted one. But it is a very nice way of releasing some energy sometimes. So it has been useful in the way of tools and it will still [continue to be useful]. Thank you.”
8. “[Referring to the theories of laughter] I didn’t realise that laughing is such a complicated business. So for me, I find that the most important thing [is] that I need to learn to laugh a bit more… to learn to relax and laugh about some of these things that I did [that] was very silly.”
9. “The chatbot is also useful because it has a lot of information. It substantiates the discussion [and gives] a lot of illustration regarding what the theory is, gives the details and then you can pick it up from there.”
10. “…I know after this course, that humour is not just an inherent property, it’s an important wisdom we should cultivate in our lives.”

The following quotes were provided by participants in a feedback questionnaire regarding their perception of the chatbot as well as its emotional impact on them from their interactions. We enumerate them here for use in discussion in the following section.

11. “Thanks to the chatbot I am more able to identify which exercise is better for me to do when I feel angry or worried.”
12. “After using the chatbot, I was usually in a good mood and happy and my bad emotions and feelings had gone.”
13. “Overall felt had a positive impact on me emotionally after having interacted with the Chatbot”
14. “It’s compassionate and polite, very nice chatbot”
15. “Using the chatbot cheered me up while doing the exercises and make me discover different forms of humor.”
16. “So easy to use via my phone. Always felt uplifted and more positive generally after interacting with the chatbot. Would appreciate continued access to it!”

## 4 Discussion

From the results of the study and the comments made by the participants, we can infer that the self-initiated humour protocol with its developmental approach can be an effective intervention strategy to improve the capacity to be humorous and learn to laugh, an intervention which, additionally, can be conducted by interacting with an emotionally intelligent AI agent. The results of our pilot study have provided the first indications about our hypotheses that SIHP, based on SAT, can improve wellbeing, self-compassion, emotion regulation, and, to some extent, psychological resilience. This creates a basis for designing a randomised control trial to further test the above hypotheses.

To the best of our knowledge, this study was the first in laughter interventions that took a developmental approach by allowing the participants to interact with their childhood selves. It was also the first study with a set of explicit rules for humour based on the main theories of laughter. While the “7 humour habits” programme presents seven general steps, the humour rules in SIHP are specific, explicit and their rationale is based on well-established humour theories and philosophies. Finally, our study, to the best of our knowledge, was the first to employ a chatbot based on the algorithmic nature of SIHP to guide the user to learn to laugh.

We can make a basic comparison between our study and the first VR-based SAT study as reported in (23) without actually drawing any definite conclusions. Both studies recruited in the non-clinical and subclinical populations (scores below 15 in PHQ-9 and GAD-7) and in both the participants interacted with their childhood avatars in an immersive VR environment using a Google Cardboard device. While the generic SAT protocol in the previous study has several laughter exercises in the second half of the 8-week course, the present study was focused on laughter for six weeks, after the first two weeks of core SAT exercises. The current study required daily practice of the exercises for 20 min at least, whereas the previous study required a minimum of 15 min twice a day. A key difference was the use of the rule-based chatbot in the current study, with which the participants could interact after the first two weeks, i.e., for six weeks in total. This chatbot had no counterpart in the previous study.

We can finally compare the effect sizes of the two studies, where the same measure was used. We note that in some cases, even though the same questionnaire was employed in the two studies, one study used Cohen’s d while the other used Matched-pairs rank-biserial correlation r, because normality was not satisfied in the data. The effect sizes we obtained for wellbeing, as measured by PERMA profiler (ours *large* d=0.97 vs. *large* d=0.86) and psychological capital measured by CPC-12R (ours *medium* d=0.73 vs. *medium* d=0.59) at the post-intervention phase were in fact greater than those obtained in the generic (N = 22) VR-based SAT pilot study (23). Similarly, we obtained a larger effect size for self-compassion, as measured by Sussex-Oxford compassion for Self Scale (SOCS-S) (ours *large* d=0.93 vs. *medium* d=0.78) at the 3-month follow-up phase.

The previous study has also reported effect sizes for a subclass of 18 compliant participants (out of 22) who had filled out a minimum acceptable dose of their diary. Comparing our results to those of the (N = 18) compliant subgroup from previous work (23), we again yield large effect sizes for wellbeing (ours *large* r=0.97 vs. *large* r=0.92) and psychological capital (ours *medium* d=0.73 vs. *medium* d=0.60) at the post-intervention phase, and larger effect size in self-compassion (ours *large* d=0.93 vs. *medium* d=0.78) at the 3-month follow-up phase. We cannot make any further direct comparisons between the results of our two studies due to the different effect size measures.

Additionally, we obtained a large effect size for the re-appraisal component of the Emotion Regulation Questionnaire (d=0.87) and participants’ use of self-enhancing humour as measured by the Humour Styles Questionnaire (d=0.80).

The quotes by participants numbered 1, 3, 5, 6, and 7, presented in Section 3.4), show that the interaction with their childhood selves as required in the protocol played a fundamental role in changing their viewpoint about life and humour. The quotes numbered 2, 4. 5, 6 and 7 showed that some participants were able to take a more light-hearted attitude to what they previously perceived as a non-harmonious or bad experience or even as a trauma in life. Finally, quotes 8, 9 and 10 show some participants, influenced by the theoretical and philosophical underpinnings of the study, have adopted a more humorous philosophy of life.

All this material is relevant to what Freud discussed in his article on humour (45) about understanding the mindset of the humorist, who “refuses to be hurt by the arrows of reality or be compelled to suffer.” Freud explains this invulnerability by comparing it to a child’s attitude: “Look here! This is all that this seemingly dangerous world amounts to. Child’s play—the very thing to jest about!” In his article, somewhat reluctantly, Freud attempted to develop a theoretical model for such an attitude by attributing it to the demands of the superego, one of the three structures in his theory of psyche. However, as explained in (11), Freud’s insight regarding this dialogue in the mind of the humorist fits much better with the interaction between the adult self and the childhood self in SAT: old childhood beliefs are revised to allow a more playful and humorous interpretation of events originally experienced as painful. We therefore submit that, given the large effect sizes we obtained, our developmental approach to learning to laugh, which challenges deeply held beliefs non-conducive to humour, is a new theoretical contribution to humour studies.

Recalling that at the outset participants were required to set a non-materialistic “noble” or altruistic goal, we can further speculate that this pursuit, throughout the SIHP engagement, contributed to a broader sense of wellbeing or flourishing. Consistent with this conjecture, the experience of compassion training (which SAT/SIHP effectively channels to the individual themselves) has been shown to enhance altruistic behaviour and the engagement of neural systems implicated in understanding the suffering of other people, executive and emotional control, and reward processing (56). Given that enhanced cognitive and emotional control can enhance goal pursuit, we can consider a “virtuous circle” where positive affect increases the salience of goals and their subsequent attainment fosters eudaimonic experience.

Another key theoretical contribution is the algorithmic nature of the SIHP, with each rule explained by one or more theories or philosophies of laughter. Based on our own mindset and interpretation of the world, these wide-ranging rules enable us to look for all the different contexts we can be humorous in a non-hostile way. This novelty addresses and provides a solution to the problem of the “black box” for comedy interventions, as described in the review article (9). The algorithmic framework also makes it possible for a chatbot to interact with and guide users to appreciate and practice the set of rules for humour based only on how they think and perceive the world. We also submit that these rules are essentially cross-cultural. While there are cultural sensitivities and variations about humour, it has been proposed that there is a certain universality for the main theories of laughter (57) and thus, we propose, for the rules derived from them.

We note from the qualitative feedback on the chatbot collected from participants that the overall impression of the chatbot and its use in delivering SIHP intervention was highly positive. (The full list of participant comments is shown in the Supplementary Material.) Participants praised the light-hearted tone of the chatbot and its use of humour sporadically throughout the conversations, citing it as having a generally positive effect on their engagement with the chatbot. This is corroborated by the results of the quantitative evaluation (Figure 3) and is in line with the findings of previous works investigating the effects of the inclusion of humour in chatbots (34, 35). It was also reported by participants that the function of the chatbot in providing protocol-relevant guidance and information had been beneficial to their understanding of the exercises and made practising them ‘more straightforward’. This sentiment can be seen reflected in the quantitative evaluation (Figure 4), where a majority of the participants reflected that they found the chatbot conversation to have flowed well and was engaging.

On the emotional impact of the chatbot, many participants reflected that the chatbot was able to have a positive impact on their emotional state immediately following their interaction, as shown in quotes 11, 12, 13, 14, 15, and 16, presented in Section 3.4. This effect was not only attributed to the aforementioned use of humorous tone but also to the perceived empathy of the chatbot, whereby participants felt the chatbot was able to respond appropriately to their emotional state (Figure 3). The quotes from participants numbered 11, 12, 13 and 14 indicate that the chatbot’s ability to provide the user with appropriate guidance, when it recognises that the user is in a negative emotional state, can be effective in helping the user to overcome their negative emotions. Lastly, we note that the majority of participants reflected in their feedback (Figure 5) that the chatbot was able to have a positive effect on their ability to recognise contexts for laughter in everyday life and was effective in aiding their learning of the protocol.

Nevertheless, there are several key limitations of our study. First and foremost, the study was not an RCT and did not have a control group, which makes its findings only preliminary. However, since this was the first human trial to evaluate SIHP, it was sensible to examine its feasibility before undertaking an RCT. Given the positive results we obtained, such an RCT can be designed and organised in the near future.

Another key limitation is that our sample population mixed non-clinical (with GAD-7 and PHQ-9 scores below 5) and subclinical populations (with GAD-7 and PHQ-9 scores at or above 5 and below 15). This precludes the evaluation of SIHP separately on the non-clinical population and on the subclinical population. In future studies, one needs to examine the impact of the protocol separately for these two different populations.

Finally, the performance of the rule-based chatbot employed in the study, while guaranteed to be non-toxic and non-hallucinating, had the serious limitation that it could not take on any open-ended conversations. This problem significantly impacted the chatbot evaluation by the participants and can potentially explain the comparatively lower agreement in evaluating the quality of chatbot conversation shown in Figure 4. Several participants explicitly pointed out that the use of an LLM would be preferred, as it would increase engagement with the users. While pointing out that the rule-based approach has been used by other similar applications of chatbots in mental healthcare (35, 37), we think in a future study, one has to undertake the challenge of training an LLM to deliver SIHP in a safe, non-toxic and non-hallucinating, as well as humorous manner. To this end, we consider Retrieval-Augmented Generation (RAG) systems (58) to be a promising compromise between strictly rule-based and fully open-ended approaches. The improving multilingual capabilities of recent LLMs also provide the potential for a scalable means to extend the delivery of SIHP to additional languages beyond English. Additionally, while a majority of our study participants found the support of the chatbot to be positive, there was a very small number of participants who reflected that they did not find the jokes from the chatbot to be humorous. This is indicative of a fundamental limitation of a rule-based approach and the subjectivity of humour, as not all jokes will be perceived as funny by everyone; means of mitigating this effect should be explored in future work to further improve the emotional intelligence of the chatbot and provide a more customisable experience.

## Data Availability

The raw data supporting the conclusions of this article will be made available by the authors, without undue reservation.

## Footnotes

^1^ https://www.prolific.com/
