# Supplementary figures and images for: Self-initiated humour protocol: a pilot study with an AI agent
Source: Front Digit Health. 2025 Mar 13;7:1530131. doi: 10.3389/fdgth.2025.1530131 (PMC11965911; doi:10.3389/fdgth.2025.1530131)

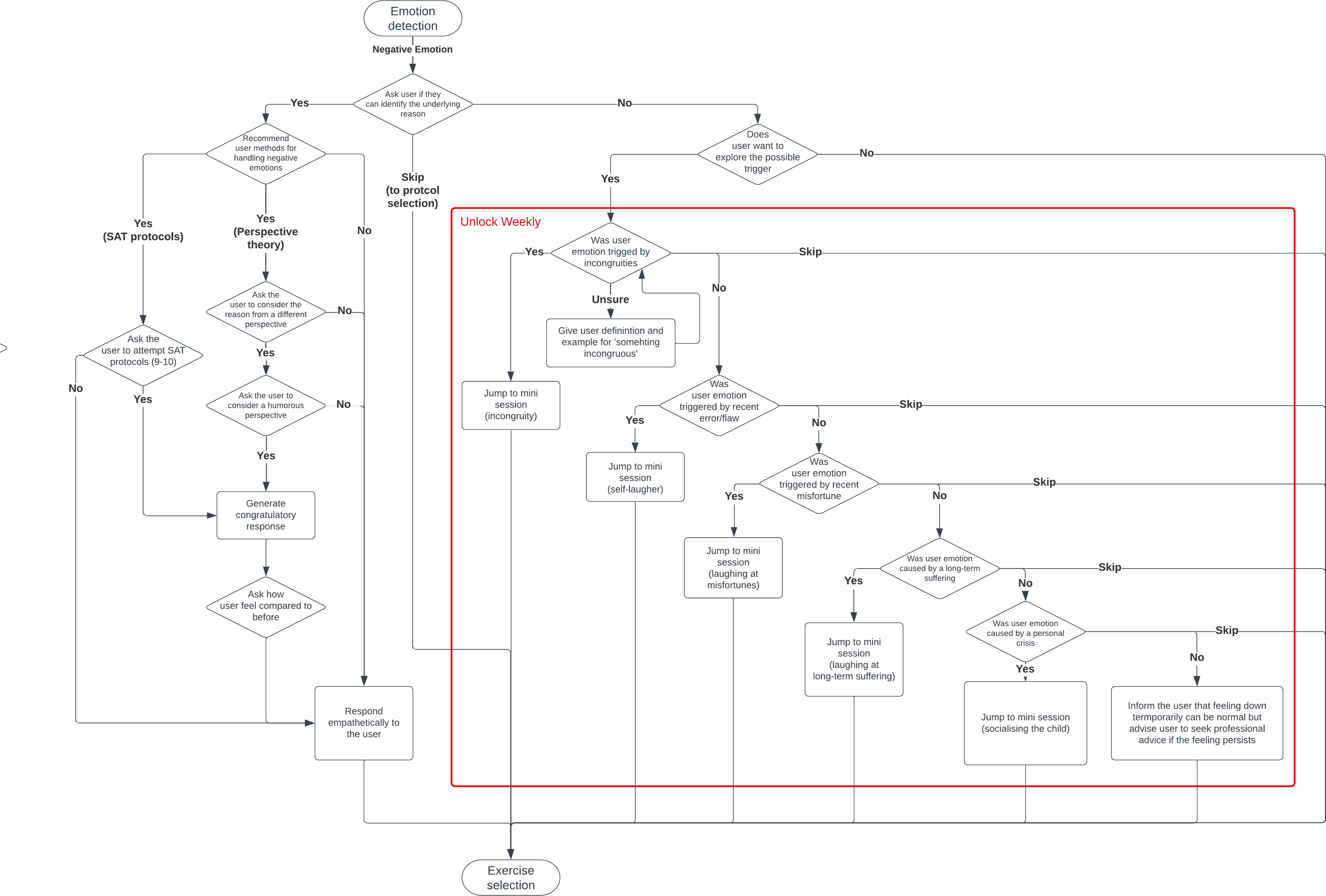

Supplement: Supplementary file 3 [file Image1.png]

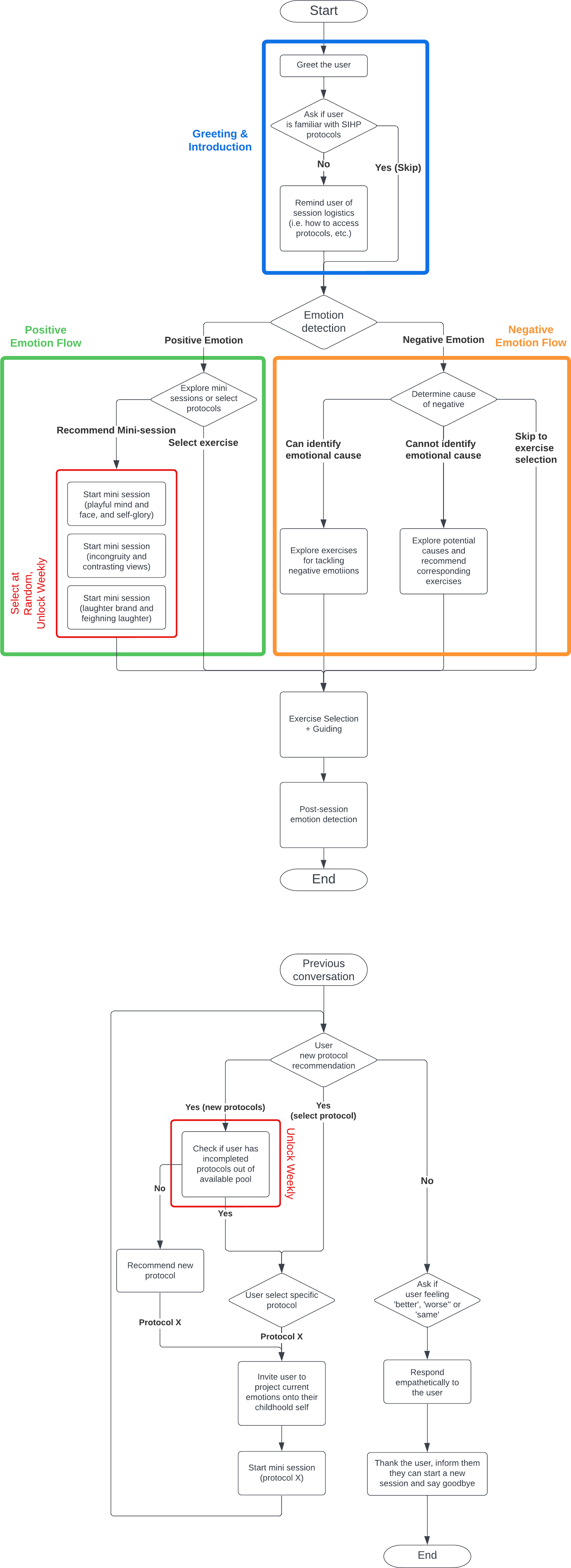

Supplement: Supplementary file 4 [file Image2.png]
